# Role of Hydrogen Sulfide in Inflammatory Bowel Disease

**DOI:** 10.3390/antiox12081570

**Published:** 2023-08-06

**Authors:** Nathalie Stummer, René G. Feichtinger, Daniel Weghuber, Barbara Kofler, Anna M. Schneider

**Affiliations:** 1 Department of Pediatrics, University Hospital of the Paracelsus Medical University, 5020 Salzburg, Austria; n.stummer@salk.at (N.S.); r.feichtinger@salk.at (R.G.F.); d.weghuber@salk.at (D.W.); b.kofler@salk.at (B.K.); 2 Research Program for Receptor Biochemistry and Tumor Metabolism, Paracelsus Medical University (PMU), 5020 Salzburg, Austria

**Keywords:** hydrogen sulfide (H_2_S), inflammatory bowel disease (IBD), Crohn’s disease (CD), ulcerative colitis (UC)

## Abstract

Hydrogen sulfide (H_2_S), originally known as toxic gas, has now attracted attention as one of the gasotransmitters involved in many reactions in the human body. H_2_S has been assumed to play a role in the pathogenesis of many chronic diseases, of which the exact pathogenesis remains unknown. One of them is inflammatory bowel disease (IBD), a chronic intestinal disease subclassified as Crohn’s disease (CD) and ulcerative colitis (UC). Any change in the amount of H_2_S seems to be linked to inflammation in this illness. These changes can be brought about by alterations in the microbiota, in the endogenous metabolism of H_2_S and in the diet. As both too little and too much H_2_S drive inflammation, a balanced level is needed for intestinal health. The aim of this review is to summarize the available literature published until June 2023 in order to provide an overview of the current knowledge of the connection between H_2_S and IBD.

## 1. Introduction

In 1996, the discovery of its neuromodulating effects changed the image of hydrogen sulfide (H_2_S) from that of a toxic gas to that of an important messenger in cells [1,2]. Since then, many important physiologic functions of H_2_S have been discovered, which is why it now belongs to the family of gasotransmitters [3]. These physiologic functions range from decreasing blood pressure to controlling the nervous system [4,5,6]. Like the other gasotransmitters, nitric oxide (NO) and carbon monoxide (CO), H_2_S is able to cross cell membranes independently of transporters or membrane receptors [7,8].

**Figure 1:**
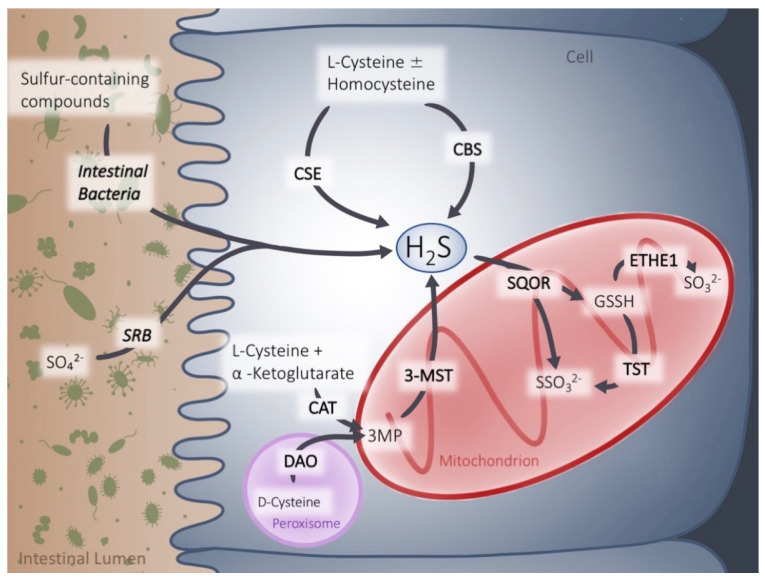
Hydrogen sulfide (H_2_S) production and detoxification. Exogenous H_2_S is a metabolic product of the degradation of sulfate (SO_4_^2−^) through sulfate-reducing bacteria (SRB) or degradation of sulfur-containing compounds by intestinal bacteria. Endogenous H_2_S results from degradation of L-cysteine with or without homocysteine by cystathionine-γ-lyase (CSE) and cystathionine-β-synthase (CBS) in the cytosol and from 3-mercaptopyruvate (3MP) by 3-mercapto-sulfurtransferase (3-MST) in mitochondria. The detoxification process is catalyzed by sulfide:quinone oxidoreductase (SQOR) and subsequently by thiosulfate sulfurtransferase (TST) or ethylmalonic encephalopathy 1 protein (ETHE1). Detoxification occurs solely in the mitochondria. Cysteine aminotransferase (CAT), D-amino acid oxidase (DAO), glutathione persulfide (GSSH), thiosulfate (SSO_3_^2−^), sulfite (SO_3_^2−^).

H_2_S can be the result of endogenous production or a product of microbial metabolism (Figure 1). The intestinal epithelial cells are exposed to both, which is why the intestine must be efficient in regulating the H_2_S concentration [9]. Endogenous H_2_S can be produced through the desulfhydration of L-cysteine with or without homocysteine, and from 3-mercaptopyruvate (3MP), produced from cysteine and α-ketoglutarate [10,11]. These reactions are catalyzed by cystathionine-γ-lyase (CSE) and cystathionine-β-synthase (CBS), as well as 3-mercapto-sulfurtransferase (3-MST), respectively [10,12,13]. Normally, the majority of H_2_S is produced from cysteine and approximately a third from homocysteine [12]. While CBS and CSE seem to be the most important H_2_S-producing enzymes, 3-MST might be more important in the colon [14,15,16,17,18,19]. Exogenously produced H_2_S is the result of microbial metabolism degrading proteins into amino acids, with cysteine and other sulfur-containing compounds among them [9,20]. Several bacterial groups, like *Fusobacterium, Clostridium, Escherichia, Salmonella, Klebsiella, Streptococcus, Desulfovibri*, and *Enterobacter*, are able to metabolize cysteine and create H_2_S in the process [21,22,23]. In particular, cysteine desulfhydrase-containing bacteria, which belong to the *Clostridium* cluster, XIVa, are abundant in the group of high H_2_S-producing bacteria that can be found in fecal samples [24].

H_2_S can be removed from the body in three ways: oxidation, methylation and expiration. Methylation and expiration, however, contribute only minimally to H_2_S disposal [25]. H_2_S is oxidized by sulfide:quinone oxidoreductase (SQOR), a mitochondrial enzyme on the inner membrane [26]. Then, either ethylmalonic encephalopathy 1 protein (ETHE1, also known as persulfide dioxygenase) or thiosulfate sulfurtransferase (TST, also known as rhodanese), other mitochondrial enzymes, further metabolize H_2_S to produce sulfate, which can exit the body in the urine via the kidneys [27,28,29,30,31,32].

Along with its physiological functions, which are still being discovered, H_2_S is gaining more and more attention as an influencing factor in many pathologies [33,34,35,36]. As the intestinal tract is exposed to more H_2_S than most other organs, many studies are focusing on the role of H_2_S in intestinal diseases, particularly its role in the not-yet well known pathogenesis of inflammatory bowel disease (IBD) [9,14,15,37,38,39,40].

IBD describes two chronic intestinal diseases: ulcerative colitis (UC) and Crohn’s disease (CD). These differ in clinical, microscopic, macroscopic, and radiological features, and can affect different parts of the gastrointestinal tract [41]. However, if the inflammation pattern does not fit into any of these groups, it is classified as indeterminate colitis (IC, or IBD unclassified (IBD-U)) [42,43]. CD is characterized histopathologically as a patchy transmural inflammatory “skip lesions”, which might appear anywhere along the gastrointestinal tract and show inflamed areas next to uninflamed ones, granulomas and deep-penetrating ulcers. Complications can be fistulas, strictures, and abscesses [44,45,46]. UC is restricted to inflammatory lesions in the colonic mucosa [45]. In CD, the terminal ileum is the most likely affected location, whereas the distal rectum is mainly affected in UC [46,47]. Both CD and UC most commonly have a clinical course of recurrent flares and remission [48].

IBD affects males and females equally, with a peak incidence between 10 and 30 years and then again between 50 and 60 years [49]. The number of people affected is rising globally, particularly in newly industrialized countries. This phenomenon is amplified by the increasing rates of diagnosis and decreasing rates of mortality [50]. About 25% of cases of newly diagnosed IBD occur during childhood and adolescence, where it tends to be more aggressive [51,52]. Many studies have shown an increase in pediatric IBD incidence in the past 20 years [53,54,55,56]. CD seems to occur earlier in the patient’s life than UC [57].

The exact pathogenesis of IBD is still unknown. Currently, it is hypothesized that a combination of genetics, environmental factors, intestinal microbiota, and changes in the immune response are responsible for triggering the onset of the disease [58,59,60,61]. There is an increasing number of genes that are linked to the disease pathogenesis. However, these are only increasing the susceptibility and are only explaining around 25% of heritability [59]. Therefore, it is proposed that interactions among genes and their products are an important factor for the onset of the disease [62]. Additionally, environmental factors, like drugs, diet, smoking, stress and geographical factors, are shown to influence the onset of IBD [63]. Furthermore, changes in the gut microbiota are observed in IBD patients, even though only 20–30% of these bacteria can be cultured [64]. Not only are there changes in the composition of the microbiota, but also in the mucus layer, in which the microbiota is located [65,66]. Lastly, immunologic factors have been suspected to play a role in the pathogenesis of IBD for the longest time. Alterations in the immune response of IBD patients have been observed in innate and adaptive immunity. Interestingly, genetic alterations, environmental factors, and the microbiota are all observed to affect the immune response too [59]. H_2_S can affect many of these factors, as it has both pro- and anti-inflammatory effects, which is why its influence in IBD is not yet well determined [15]. The following review will focus on the crucial role of H_2_S in the pathogenesis of IBD.

## 2. Materials and Methods

This review was conducted in line with the Preferred Reporting Items for Systematic Reviews and Meta-Analysis (PRISMA) guideline [67].

### 2.1. Data Source/Search Strategy

Research for studies on the connection of H_2_S and IBD was conducted on PubMed with the use of the following search terms: H_2_S, hydrogen sulfide, IBD, inflammatory bowel disease, Crohn‘s disease, ulcerative colitis, chronic inflammatory disorder of the bowel, gastrointestinal tract, colonic mucosa, intestinal barrier dysfunction, SRB, sulfate-reducing bacteria, H_2_S metabolism, cystathionine-γ-lyase, CSE, cystathionine-β-synthase, CBS, 3-mercapto-sulfurtransferase, 3-MST, sulfide:quinone oxidoreductase, SQOR, thiosulfate sulfurtransferase, TST, and ethylmalonic encephalopathy 1 protein ETHE1. The results included studies from July 1967 to June 2023.

### 2.2. Study Selection

The articles listed on PubMed were first screened based on titles and again after reading the abstract. The list of references in the selected papers was then examined for other studies which were relevant for this review. All studies written in English and listed on PubMed were included in the screening, independently of the study design. 

The references in this review were extracted from Endnote software version X9 (Thompson Reuters; New York, NY, USA). 

**Figure 2:**
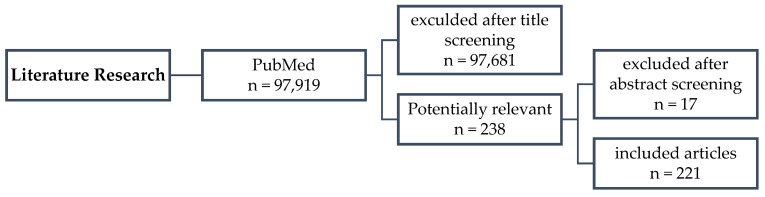
Flow chart of the screening and selection process.

The selection process is visualized in Figure 2.

## 3. The Connection between H_2_S and the Pathogenesis of IBD

### 3.1. Microbial H_2_S

In the gastrointestinal tract, the number of bacteria is higher than in any other ecosystem [68]. There they form the microbiota, among which the many functions are the degradation of otherwise undigestible food components, e.g., complex carbohydrates [69]. The result of this metabolic processes are short-chain fatty acids, which are an important energy source for colonocytes [70]. Microbial degradation of sulfur-containing amino acids, on the other hand, produces H_2_S [71]. Another important function of the microbiota is the defense against pathogenic and opportunistic microorganisms. An imbalance in this microbial system is linked to certain diseases, e.g., IBD, cancer and diabetes [68,72]. These diseases might also be linked to H_2_S [68,73]. The crucial role of bacteria in the microbiota in the pathogenesis of IBD is also seen in animal models, in which antibiotics or a germ-free environment ameliorate an inflammation of the colon [74]. In bacteria, sulfide can either be the result of degradation of sulfate-containing substances or as-/dissimilatory reduction of sulfate or sulfur [20,71,75]. Since transit time and other factors, like pH, determine which metabolizing pathways can be active, the majority of bacterial sulfide production is located in the large intestine [20]. The substrate for bacterial H_2_S production can either be dietary or endogenous [76,77]. In the large intestine, the endogenous colonic mucins are much more sulfated than in the small intestine, providing a great supply for H_2_S production and therefore an optimal environment for H_2_S-producing bacteria [78].

H_2_S can be produced by sulfate-reducing bacteria (SRB) in the colonic lumen. A change in the microbiota is seen in patients with CD and UC compared to healthy ones, e.g., a higher level of activity of SRB is suggested in the colon of UC patients [79,80]. Higher levels of H_2_S and SRB are found in stool samples from patients with UC [38]. Likewise, a decrease in butyrate, acetate, methylamine, and trimethylamine, all products of typical gut bacteria, in fecal extracts of IBD patients reinforces the idea of a change in the microbiota [81]. Higher level of SRB correlated with the symptom severity of IBD. Accordingly, in patients with active disease, more SRB were detected than in patients in remission [82]. 

In contrast to the increased numbers of SRB, the autochthonous bacterial flora seems to be reduced in UC patients [83]. The higher temperature, created by the inflammatory process, increases the growth of some SRB, which then also increases H_2_S production [83]. SRB produces acetate, which in turn inhibits the enzyme TST. This leads not only to a decrease in the detoxification of H_2_S but also to an increased permeability of H_2_S through the intestinal barrier [84]. This synergy of H_2_S and acetate intensifies the aggressiveness of H_2_S in the colonic lumen [83]. 

The microbiota of children with IBD is altered in contrast to that of healthy ones. The changes differ from the alterations seen in adults. Here, different bacterial groups predominate, like Faecalibacterium prausnitzii, which is found to be decreased in adults with IBD [85,86,87]. These studies used culture-based technology to examine the configuration of the microbiota. As not all bacteria in the microbiota can be cultured, a comprehensive molecular analysis of the pediatric microbiota in IBD patients is needed [64,88]. Nevertheless, the number of H_2_S-producing bacteria is found to be increased in children as well as in adults with new-onset IBD [39].

In pediatric CD patients, a decrease in butyrate, a short-chain fatty acid, which is not only an important energy source but also has anti-inflammatory properties, is seen [39,89]. Furthermore, butyrate is shown to improve the barrier function [90]. Butyrate seems to stimulate mitochondrial gene expression as well as mitochondrial H_2_S-detoxification enzymes [39,91]. The decrease in butyrate might be due to a decrease in butyrate-producing bacteria, which is seen in the microbiota of pediatric CD patients [39]. This decrease in butyrate—and consequently in the mitochondrial proteins that are less expressed—seems to decrease the ability of H_2_S-detoxification [39]. Additionally, butyrate lowers the level of epithelial oxygenation [92,93]. H_2_S, on the other hand, increases the level of oxygen through the inhibition of β-oxidation [94]. This combination establishes a hostile environment for obligate anaerobe bacteria, which produce short-chain fatty acids [95]. A reduction of butyrate-producing bacteria is also linked to antibiotic exposure, which in turn is suspected to increase the risk of pediatric CD [39,89,96]. The production of H_2_S seems to be a defense mechanism against antibiotics, which favors SRB growth in the colon and might therefore be partly responsible for the increased risk [97,98]. In addition, the lack of short-chain fatty acids increases the pH, which favors the growth of SRB even more [95]. 

The decrease in butyrate combined with the ability of H_2_S to inhibit the cytochrome c oxidase in mitochondria and β-oxidation may result in energy starvation and oxidative stress, which damages colonocytes and subsequently the gut barrier [95,99]. This allows intestinal microbes to get into direct contact with the mucosal immune system, which then gets activated, resulting in inflammation [95]. This creates a vicious cycle: the inflammation further damages the epithelial barrier, driving further activation of the immune system and resulting in more inflammation [95,100].

Another factor that links the decrease of butyrate with H_2_S is the availability of H_2_. H_2_ is crucial for anaerobic respiration in the intestinal lumen. One of the bacterial groups that need H_2_ for anaerobic respiration are SRB [101]. H_2_ is the product of intraluminal carbohydrate fermentation by bacteria [102]. In the event of too little production, e.g., low dietary intake of carbohydrate that can be fermented to H_2_, NADH is consumed as an alternative source of H_2_, which is used for H_2_S-production at the cost of butyrate production [95,103].

In stool samples from IBD patients, a higher amount of metabolites containing sulfur compared to stool from healthy individuals is seen, which might indicate a dysfunction in the metabolism of H_2_S [104,105]. The cause for this is most likely multifactorial, with changes in diet and therefore in the availability of substrate and alterations in the synthesis or metabolism of H_2_S produced from the cells themselves or bacteria in the microbiota being some of these factors [105]. Part of the increase in these sulfur-containing metabolites might originate from the increase in taurine. Taurine can result from the hydrolyzation of taurine-conjugated bile acids. However, the activity of bile salt hydrolase is seen to be decreased in active IBD [106,107]. One of the most important bile acid hydrolyzers in the gastrointestinal tract is from the Firmicutes phylum, which is reduced in IBD patients [106,108,109]. The highest activity of bile salt hydrolase is seen in Lactobacilli [110]. In patients with UC, reduced numbers of lactic acid bacteria are found [111]. Nevertheless, Kushkevych et al. showed a reduction in lactic acid bacteria levels correlating with increased levels of H_2_S [112]. Other bile salt hydrolyzers are also found to be decreased in IBD patients [106,113]. The increase in the availability of taurine might therefore be due to the promotion of endogenous taurine production by increased levels of methionine and cysteine from diet or from a dysfunctional cysteine or methionine metabolism as seen in IBD [105]. Taurine, when metabolized by bacteria in the gut, results in increased substrate for H_2_S production [105]. *E. coli*, which commonly metabolizes taurine, among others, is found to be increased in IBD [114,115]. The abundance of bile acids in combination with the increased availability of methionine and cysteine in IBD could therefore promote the occurrence of certain bacteria, which consequently increase the level of H_2_S [105].

A common drug in the treatment of IBD, 5-aminosalicylic acid (mesalamine), suppresses the growth of SRB and consequently decreases the level of produced sulfide, indicating another possible mechanism for its beneficial effects [82,116]. Additionally, mesalamine, like butyrate, stimulates the peroxisome proliferator activated receptor gamma (PPAR-γ) [117,118]. This receptor is responsible for the promotion of β-oxidation and lowers the oxygen levels of the epithelium, which in turn drives the growth of obligate anaerobes. These bacteria produce butyrate and therefore provide energy to the colonocytes [119]. However, an even more efficient decrease in inflammation and especially a decrease in nociception, which is absent with 5-aminosalicylic acid, is seen in a study with ATB-429, a H_2_S-releasing derivate of mesalamine [120]. Nevertheless, decreasing sulfide levels by removing the substrate for its production from SRB through a reduction in sulfur-containing amino acids ameliorates colitis as well [121,122]. An increased abundance of SRB and consequently H_2_S is also linked to other inflammatory diseases like periodontitis and pouchitis [98,123,124]. 

A decrease in the number of SRB might be one possible explanation for the beneficial effect of an appendectomy in UC [125]. In a healthy state, the appendix harbors many gut microbes, one of which is *Fusobacterium* spp., which is an efficient producer of H_2_S [126,127]. This genus of bacteria is increased in an inflamed appendix [128]. Consequently, an appendectomy and therefore the removal of a source of H_2_S production, may be a reason for the preventive effect of UC [95].

Nevertheless, some studies found no change in the luminal H_2_S of IBD patients and indicate that the bacterially produced H_2_S might be bound and therefore metabolically inactive [129,130]. However, the accuracy of the estimates of luminal H_2_S has been questioned [14]. The analysis of fecal samples is limited since it examines only the end product and therefore only the metabolizing processes at the end of the gastrointestinal tract [100]. Additionally, the colonic transition rate of feces influences the fecal H_2_S levels, resulting in lower accuracy of the estimates of luminal H_2_S [131].

In colon samples from healthy individuals, the microbiota is organized in biofilms, which form when colonies encapsule themselves in secreted polysaccharides. This supports them in the harsh living conditions they face in the gastrointestinal tract [132,133]. In control colon samples from mice and rats, these biofilms form on the sterile mucus layer. After induction of colitis, they were disorganized with bacterial translocation into the lamina propria [40]. Likewise, in healthy humans, the bacteria from the microbiota have no contact with the intestinal epithelium because of these biofilms [66,134]. In IBD patients, however, this is not the case [135,136,137]. Here, the bacteria actually do have contact because of an even thinner and more discontinuous mucus layer, which results in less epithelium covered than in healthy colons [137,138,139]. In UC patients, the mucus is even seen to be more permeable and less viscous [122,140]. This results in decreased barrier function, which is also suspected to play a significant role in the pathogenesis of IBD [141]. The changes in mucus permeability approach normal levels when IBD patients are in remission [136]. H_2_S might be able to reduce the linking of disulfide bonds in the mucus layer and allow bacteria to penetrate the remaining mucus layer, distorting it even more [142]. 

On the contrary, in animal models, it is shown that giving H_2_S donors does not elicit an inflammatory response but even helps to maintain a normal mucus and microbiota structure [40,143]. H_2_S is an important stimulant for mucus production in the colon, which promotes the establishment of a microbiota biofilm [144]. During inflammation of the colon, mucus production is reduced, which leads to fragmentation of the otherwise linear biofilms. This effect is reversed by administration of H_2_S donors [40]. Healing of the tissue damaged by colitis is delayed by inhibiting the synthesis of H_2_S and accelerated by adding H_2_S donors [14,19]. 

The beneficial or destroying effects of H_2_S on the mucus might therefore be concentration dependent, too, and underline the assumption of H_2_S having a dome-shaped curve of beneficial concentration levels in the colon, with too high and too low levels being harmful [145]. 

### 3.2. Non-Microbial and Endogenous H_2_S

The contribution of sulfur to the pathogenesis of IBD is supported by many animal models in which dextran sodium sulfate (DSS) damages the colonocytes, which allows for luminal bacteria to enter, consistently resulting in an inflammatory state very similar to that of IBD [146]. 

In the colon of rats, intraluminal exposure to NaHS, a salt that eventually forms H_2_S, even for a short period of time, leads to an inflammatory response. The rise in genetic expression interleukin-6 (IL6) is only seen with high concentrations of NaHS; a lower concentration does not seem to elicit an inflammatory response. In those rats as well as in human colonocytes, DNA is not harmed by NaHS. However, it seems to put colonocytes in a hypoxia-like state by inhibiting mitochondrial oxygen consumption. This inhibitory effect is seen at high concentrations of NaHS, whereas low concentrations seem to stimulate oxygen consumption. This leads to the assumption that higher luminal H_2_S concentrations have a negative effect on colonocytes due to the inhibition of oxygen consumption and, in turn, of the energy metabolism of this highly energy-demanding tissue, as well as due to the elicitation of inflammatory gene expression [147]. However, this seems to be a reversible effect [147,148]. The inhibition of oxygen consumption can be reduced by dietary proanthocyanidin-containing polyphenols in different fruit extracts. It is suggested that these polyphenols can bind H_2_S [149]. This H_2_S-binding characteristic is also seen with zinc chloride [150]. 

CSE, CBS, and 3-MST are endogenous enzymes that produce H_2_S [10,12,13]. SQOR, ETHE1, and TST are responsible for the detoxification process [26,27,28,29,30,31,32]. Some studies have shown differences in the enzymatic expression and the presence of enzymes involved in the metabolism of H_2_S in inflamed tissue and healthy controls [14,16,17,39,100,145,151,152] (Table 1). Hirata et al. demonstrated an increase in mRNA levels of CSE and CBS and, consequently, in H_2_S levels in the colonic mucosa of mice with DSS-induced colitis. In this animal model, the CSE seems to be the dominant producer of H_2_S, as CSE mRNA levels were more than 200 times higher than those of CBS [151]. De Cicco et al. showed decreased expression of CBS [16]. Wallace et al., however, concluded that the observed increase in the capacity of H_2_S production in rats with colitis is due to an increase in activity rather than expression, which was decreased overall [14]. In IBD as well as in in vitro inflammatory settings, 3-MST is reduced and in an animal model with complete deficiency of this enzyme, the inflammation, based on the level of inflammatory cytokines, the clinical symptoms and the histological findings, is seen to be more extensive than in the wild-type counterpart. The level of ROS and consequently intestinal epithelial cell apoptosis also correlated with 3-MST expression [153]. Interestingly, providing NaSH as an external source of H_2_S does not ameliorate the intestinal inflammation in 3-MST-deficient mice. This study suggests that the influence of 3-MST on AKT signaling, a regulator for epithelium inflammation, epithelium apoptosis, cell proliferation and dendritic cell maturation, and not the changes in H_2_S, is responsible for 3-MST-mediated colitis [153].

The detoxification of H_2_S also seems to be altered in IBD. Activity and expression of rhodanese are significantly reduced in DSS-induced colitis [17]. De Preter et al. also reported a decrease in TST enzyme activity and gene expression in tissue samples from UC patients [100]. This is in contrast to the study by Picton et al., which showed no difference in the activity of this enzyme in patients with IBD. The samples used, however, were taken from patients who had already been on medication and only rectal biopsies were used [152]. Other H_2_S-detoxification enzymes, namely ETHE1 and SQOR, are downregulated in the colon of patients with CD. This study, again, showed a repression of TST [39]. Another study, which examined all enzymes except CBS, showed a decrease in all enzymes in intestinal epithelial cells of adult IBD patients when compared to healthy adults by immunohistochemical examination of intestinal samples. Interestingly, in children with IBD, there is not such a pronounced difference in enzyme expression in epithelial cells [145].

In UC and CD patients, the gene expression, as well as the enzyme activity of the detoxification enzyme TST are reduced [100,154]. In CD patients, the reduction is less extensive after therapy with anti-tumor necrosis factor α-therapy with infliximab [154]. In these studies, a connection between inflammation and the expression of these enzymes is postulated. The authors of these studies therefore describe the changes in gene expression as a result of inflammation rather than a cause [100,154].

The reason for the contradictory data regarding the difference in enzyme expression has not been identified yet. Most studies discovered a lower expression, while only a few reported an increase in the enzyme expression [14,16,17,39,100,145,151,153].

None of the studies that showed increased expression were conducted on human samples and Wallace et al. reported in the immunohistochemical staining an increase in CSE expression in the mucosa and submucosa while the epithelial cells remained unstained [14,151]. To fully comprehend the changes in the endogenous metabolism further and more extensive studies are needed.

The crucial role of a healthy detoxification ability of the colon is confirmed in an animal study. Here, it is shown that the colonic mucosa of healthy rats absorbs up to 95% of the produced H_2_S. The amount of absorbed H_2_S would be lethal if it were to reach systemic circulation. Due to the efficient conversion of H_2_S to thiosulfate, the colonic tissue is not damaged by the high luminal H_2_S levels and the toxic amount of H_2_S is prevented from entering the systemic circulation [131]. The colonic mucosa is especially equipped for this task, as it detoxifies H_2_S much faster than other parts of the gastrointestinal tract [155].

CBS and CSE, two endogenous synthesizers of H_2_S, both require vitamin B6 as a cofactor [156]. This vitamin is deficient in nearly a third of IBD patients due to inadequate intake or absorption secondary to the inflammation [157]. Flannigan et al. showed that an induced vitamin B6 deficiency resulted in an impairment of H_2_S synthesis in the colon and worsened colitis in animal models. Consequently, a H_2_S donor ameliorated these effects. They even observed decreased colonic CSE expression in rats with vitamin B6 deficiency [156]. 

On the other hand, H_2_S provides a source of energy to the epithelial cells in the gastrointestinal tract as long as the concentration does not exceed certain levels [158]. H_2_S itself seems to have anti-inflammatory effects in low concentrations: H_2_S prevents leucocyte adherence in the vasculature [159] and inflammation-triggered plasma exudation [160]. H_2_S can also decrease pain sensation in the colon [161], and promotes healing [162] as well as resolution of colitis in preclinical animal trials [14,19,151,163]. Moreover, H_2_S protects mitochondria and their function in situations in which oxygen is low by upregulating the nuclear factor erythroid 2-related factor 2 (Nrf2) stress response pathway, which increases detoxifying proteins and antioxidants [164,165,166,167]. Endogenous H_2_S is also able to reduce inflammation by decreasing the production of pro-inflammatory cytokines and by modulating the frequency and number of granulocyte-like myeloid-derived suppressor cells [16]. Hirata et al. showed that endogenously derived H_2_S also acts as an antioxidant [151]. Furthermore, H_2_S suppresses the activation of nuclear factor kappa-light-chain-enhancer of activated B cells (NF-κB), an important regulator for the immune response, and even promotes the healing of gastric ulcers [168,169]. Giving H_2_S donors significantly alleviated colitis in rat models, whereas inhibition of H_2_S synthesis in those rats and healthy rats induced or worsened colitis. The mechanism behind this might be a decrease in cyclooxygenase-2 messenger RNA expression and the resulting decrease in prostaglandin synthesis [14]. In chronic inflammation, this is important for the resolution of the inflammatory response [144]. The exacerbation of inflammation after blocking the synthesis of H_2_S is also seen in cells with DSS-induced colitis [170].

Flannigan et al. confirmed a connection between IL-10, an anti-inflammatory cytokine, and H_2_S [156]. The initially observed reduction in H_2_S synthesis in IL-10-deficient mice was reestablished to normal levels with recombinant IL-10 [156]. H_2_S has a stimulatory effect on IL-10 production while decreasing pro-inflammatory cytokines such as IL-1β, IL-6, IL-8, IL-18, tumor necrosis factor-alpha (TNF-α) and interferon-gamma (IFN-γ) [120,171,172,173,174]. A link between IL-10 and IBD has already been shown in many studies, which confirm that impaired IL-10 secretion intensifies the inflammation in IBD [175,176,177]. IL-10 is crucial for mucosal homeostasis and regulation of the immune response in the colon [178,179]. Indeed, a mutation in IL-10 signaling, as seen in nucleotide-binding oligomerization domain 2 (NOD2) mutations, is linked to an increased susceptibility to IBD [175,180]. H_2_S thereby decreases inflammation by stimulation of IL-10. This is also observed in other organs, such as the brain, liver or lung [181,182,183]. Additionally, H_2_S protects the mucosa by increasing blood flow through vasodilation. This is important for decreasing mucosal damage through all erosive substances, like bile, acids and digestive enzymes, it faces and for fastening tissue repair after damage occurs [167,184,185,186].

This controversy in pro- and anti-inflammatory effect of H_2_S shows, again, that unbalanced levels of H_2_S—whether they are too high or too low—drive inflammation. Therefore, a certain range of H_2_S concentration level might be needed for intestinal health.

### 3.3. Dietary H_2_S

Dietary factors also seem to influence the inflammatory activity in IBD. Sulfur-containing amino acids, which can be transformed to H_2_S, can also be derived through diet [20,187]. Sulfate can be absorbed in the small intestine very efficiently; however, this mechanism has a saturation point [188]. Once this level is reached, the amount of sulfate reaching the colon increases with dietary intake. Nevertheless, other factors, like food preparation or meal consumption habits, can also influence the number of sulfates reaching the colon, making dietary factors difficult to study regarding their role on H_2_S and IBD even harder to study [95,189,190,191]. A western diet seems to be especially rich in inorganic sulfate and protein-derived sulfate [192]. A link between IBD and diet seems likely, considering the rise in IBD numbers in westernized nations. Until a decade ago, mostly Caucasian people were affected by IBD. Nevertheless, the incidence has been rising recently in the Asian and Hispanic populations, especially in those who immigrated into high-prevalence countries and particularly in their children [193]. Additionally, residents of urban centers are more at risk of developing IBD than those in rural settings [49]. Studies on the link between H_2_S and IBD are limited. Most of them focus on lowering sulfur intake by transitioning to a more plant-based diet, which seemed to ameliorate the disease activity. However, these studies are either case reports or have relatively low patient numbers [121,194,195,196,197].

Sulfate can be found in high amounts in many food additives, dried fruit, nuts, some vegetables, wheat bread, sausages, milk products, beer, canned and pickled products [68,198]. Dietary habits also have an influence on the microbiota. A diet high in protein is linked to increased numbers of SRB, which increases H_2_S production and decreases the number of butyrate producers and therefore the amount of butyrate in the colon [199,200]. These changes are also observed in patients with IBD [200,201,202,203]. A diet providing a high amount of fermentable carbohydrates leads to increased butyrate production, which subsequently lowers the pH [204,205]. A more acidic environment favors the growth of other bacterial groups, like butyrate producers, over SRB [95,204,206], while H_2_S production works best in an alkaline environment [207]. In vegans and vegetarians, a lower stool pH is found in comparison to omnivores, consistent with a more even distribution of short-chain fatty acid up to the distal end of the colon [95,208]. When feeding mice a diet with a high fat content, a reduced production of short-chain fatty acid and an increase in H_2_S production is observed [209]. In IL-10 knockout mice, which show a similar colitis to that of IBD, a diet containing a lot of saturated fat increases inflammation severely and leads, in combination with a mutagen, to the formation of adenomas [95,209,210]. All in all, a typical western animal-based diet, consisting of a high fat and protein intake, might create the perfect environment for H_2_S production at the expense of butyrate production, which subsequently drives inflammation [95]. Accordingly, many IBD patients believe that their symptomatic relief relies more on their diet than their medication [211].

This assumption is partly supported by the success of exclusive enteral nutrition (EEN) in the treatment of CD. EEN describes a strict liquid diet consisting of formula for a duration of 4 to 12 weeks [212,213]. Especially with pediatric IBD patients, EEN is shown to be as efficient as therapies with corticosteroids and to achieve remission in nearly 80% [212,214,215]. The reason for the reduction in inflammation with EEN is still mostly unknown [216]. One of its mechanisms of action could be alterations in the microbiota [217,218]. It was reported that EEN reduced the number of a very potent H_2_S producer, Atopobium parvulum [39,219]. Unfortunately, after cessation of EEN, the changes in the composition of the microbiota return to their prior state, concurrently with rising fecal sulfide levels [199,220,221].

## 4. Conclusions

The pressing need for a deeper understanding of the pathogenesis of IBD is growing with the rising numbers of people affected by this chronic disease [222]. As suspected in many other diseases, H_2_S is believed to play a crucial role in IBD [33,34,35,36]. As the intestinal tract is exposed to microbial, endogenous and dietary H_2_S and therefore, to more than most other organ systems, a change in the level of H_2_S is hypothesized to influence the health of the intestinal system [9,14,15,37,38,39,40]. Changes in the microbiota as seen in IBD, e.g., an increase in SRB and consequently a decrease in butyrate-producing bacteria, the decrease of H_2_S-detoxifying enzymes in the intestinal epithelial cells and the increasing intake of dietary sulfate by eating a typical western animal-based diet, indicate that too much H_2_S has pro-inflammatory effects in IBD. However, the restorative effects of H_2_S on the mucus barrier and microbiota biofilms, its decreasing effects on pro-inflammatory cytokines and its overall healing-promoting characteristics also suggest that too little H_2_S can be pro-inflammatory. Therefore, it is likely that there is a dome-shaped curve of beneficial concentration levels in the intestinal tract. Even though it would be essential to measure intraluminal intestinal H_2_S levels directly to determine if H_2_S could be a possible target in the treatment of IBD, all these studies definitely underline the connection between H_2_S and the pathogenesis of IBD.

## Figures and Tables

**Table 1:** Overview of studies on changes in hydrogen sulfide-producing and -detoxifying enzymes (cystathionine-β-synthase (CBS), cystathionine-γ-lyase (CSE), 3-mercapto-sulfurtransferase (3-MST), ethylmalonic encephalopathy 1 protein (ETHE1), sulfide:quinone oxidoreductase (SQOR) and thiosulfate sulfurtransferase (TST)) in human and animal samples. Ulcerative colitis (UC), Crohn’s disease (CD), dextran sulfate sodium (DSS).

Enzyme	Method	Difference in Enzyme Expression in Comparison to the Control Group	Species	Reference	Year
CBS	mRNA measurement	increased	Mouse (DSS-induced colitis)	Hirata et al. [151]	2011
CBS	mRNA and protein expression	decreased	Mouse (Helicobacter hepaticus induced colitis in mice without adaptive immune system	De Cicco et al. [16]	2018
CBS	Protein expression (Western blot)	first lower (days 3–7 after induction), then increased	Rat (trinitrobenzene sulfonic acid induced colitis)	Wallace et al. [14]	2009
CBS	Immunohistochemical staining	increased	Rat (trinitrobenzene sulfonic acid induced colitis)	Wallace et al. [14]	2009
CSE	mRNA measurement	increased	Mouse (DSS-induced colitis)	Hirata et al. [151]	2011
CSE	Protein expression (Western blot)	decreased	Rat (trinitrobenzene sulfonic acid induced colitis)	Wallace et al. [14]	2009
CSE	Immunohistochemical staining	Unstained epithelial cells, while increased staining of mucosa and submucosa	Rat (trinitrobenzene sulfonic acid induced colitis)	Wallace et al. [14]	2009
CSE	mRNA and protein expression (Western blot)	decreased protein levels, decreased mRNA	Rat and mouse (DSS-induced colitis)	Taniguchi et al. [17]	2009
CSE	Immunohistochemical staining	decreased	Human (CD + UC)	Stummer et al. [145]	2022
3-MST	Protein levels (Western blot)	decreased	Human (CD + UC)	Zhang et al. [153]	2022
3-MST	Immunohistochemical staining	decreased	Human (CD + UC)	Zhang et al. [153]	2022
3-MST	mRNA and protein expression	decreased	Mouse (DSS induced colitis)	Zhang et al. [153]	2022
3-MST	Immunohistochemical staining	decreased	Human (CD + UC)	Stummer et al. [145]	2022
ETHE1	mRNA	decreased	Human (CD)	Mottawea et al. [39]	2016
ETHE1	Immunohistochemical staining	decreased, except for the terminal ileum in pediatric patients	Human (CD + UC)	Stummer et al. [145]	2022
SQOR	mRNA	decreased	Human (CD)	Mottawea et al. [39]	2016
SQOR	Immunohistochemical staining	decreased, except for the terminal ileum	Human (CD + UC)	Stummer et al. [145]	2022
TST	mRNA and protein expression (Western blot)	decreased	Rat and mouse (DSS induced colitis)	Taniguchi et al. [17]	2009
TST	mRNA	decreased	Human (UC)	De Preter et al. [100]	2012
TST	mRNA	decreased	Human (CD)	Mottawea et al. [39]	2016
TST	Immunohistochemical staining	decreased	Human (CD + UC)	Stummer et al. [145]	2022
